# California State Veterinary Medical Association

**Published:** 1893-10

**Authors:** R. A. Archibald

**Affiliations:** Secretary


*President.*

---

*California State Veterinary Medical Association.*—A regular meeting of the California State Veterinary Medical Association was held on September 13th, 1893, at the Baldwin Hotel, San Francisco.

The meeting was called to order by the President, Dr. Wm. F. Egan.

Upon roll call the following gentlemen responded: Drs. Egan, Burns, Maclay, Spencer, Sr., Spencer, Jr., Fox, Wadams, Orvis, and Archibald. Visitors present were: Drs. Patterson, Robin, Jackson, Williams, Hagarty and Dalzeil.

The minutes of the previous meeting were read and approved.

The reading of papers, discussions, etc., brought Dr. H. A. Spencer, of San Jose, to his feet with an excellent and instructive paper on Gastro Hysterotomy. The essayist gave the history of the operation, the origin of which dated back before Christ. He then went on to describe the operation in detail, as operated by him on a bitch; he also described his after treatment, etc.

The President then declared a discussion was in order, whereupon Dr. Maclay arose and complimented the essayist on the successful issue of the operation. Drs. Fox, Spencer, Jr., and the Secretary also joined in the discussion, and gave their views on the subject. The discussion was followed by a few well-chosen remarks by the President.

The Secretary was then called upon to entertain the meeting, which he endeavored to do by reading a few notes on an operation witnessed by him. The operation was the Extirpation of a "Tuto-ovarian Cyst." The essayist described the operation as seen by him, giving the after treatment, etc., also giving a short treatise on the cause of the lesion.

The paper was followed by a discussion, in which most of the members participated. The Secretary then brought up a subject upon which he wished to obtain the view of the members. The matter was the treatment of sores which Jacks and Mules are very subject to. He described a case in a two-year old thoroughbred colt, which he was at that time treating. He gave the methods of treatment which he had applied, with results. Dr. Robin favored the use of Ung. Hyd. Nit. Dr. Williams said that he had had considerable experience with the subject under consideration, and that the most beneficial treatment he had found was Ung. Hyd. Nit. Dr. Spencer, Jr., uses Phytolacca, both externally and internally with good success. Dr. Spencer, Sr., claimed to have good results from the use of a paste compound composed of Zinc. Chl., Sanquinaria and flour. Dr. Wadams uses the actual cautery, followed by iodoform dressings. Dr. Orvis said that he never had much success with the disease, but he favored the use of the actual cautery, followed by astringents. The Secretary said that he always advised his clients who were unfortunate in possessing Jacks affected with these sores, to put them in fly tight loose boxes during the day, turning them out at night for exercise.

The Secretary then proposed the following names for membership : Drs. Paterson, Falkner, Robin, H. Fabbi, Forrest, Williams, Jackson, Hogarty and Dalzeil. The names were ordered referred to the Board of Examiners.

Under the head of new business the Secretary read a communication from the Secretary of the U. S. Veterinary Medical Association, asking the Society to appoint delegates to represent the local organization at the International Congress. The matter was discussed at some length by the Secretary, who endeavored to point out the benefits the Society would receive by sending delegates to the International Congress. He also spoke at some length on the intentions of the National Organization, and the manner in which it intended to benefit the Veterinary profession in the United States. He implored the members of the Society to join the National Organization, and pointed out the advantages they would gain by such a procedure. He said the expenses of joining the National Association were, comparatively speaking, small, and that everyone who joined would be fully repaid in the future.

On motion by Dr. Maclay, seconded by Dr. Spencer, the Secretary was instructed to write to the Secretary of the U. S. Veterinary Medical Association and thank him for his kind invitation ; also to acquaint him with the fact that the society at the present time was in a position financially to accept his invitation, as it had lately gone to considerable expense in legislative matters.

The Secretary then presented a written notice that at the next meeting he intended to move to amend the Constitution and By-laws, as owing to the passage of an Act entitled "An Act to regulate the practice of Veterinary Medicine and Surgery in the State of California," at the last Legislature, some changes in the Constitution and By-laws were necessary. The notice was ordered referred to the Board of Directors.

Nomination for officers for the ensuing year were declared in order by the President. The nominations resulted as follows: For President, Dr. H. A. Spencer; Vice-President, Dr. W. B. Rowland; Secretary, no nomination; Treasurer, Dr. D. F. Fox; Board of Examiners, Drs. Maclay, Egan, Orvis, Rowland, and Whittlesey. Board of Directors, the several officers of the Association. On motion by the Secretary the name of J. C. C. Price was dropped from the roll of membership.

On motion by Dr. Wadams a vote of thanks was tendered to the essayists for the able and masterly manner in which they had entertained the meeting.

The following gentlemen were appointed essayists for next meeting: Drs. Maclay, Orvis and Archibald.

There being no further business before the meeting, it adjourned to meet in Sacramento, on Dec. 13th, 1893.

R. A. ARCHIBALD, D.V.S.,
*Secretary.*

